# Real life Experience of Medical Cannabis Treatment in Autism: Analysis of Safety and Efficacy

**DOI:** 10.1038/s41598-018-37570-y

**Published:** 2019-01-17

**Authors:** Lihi Bar-Lev Schleider, Raphael Mechoulam, Naama Saban, Gal Meiri, Victor Novack

**Affiliations:** 1 Clinical Cannabis Research Institute, Soroka University Medical Center and Faculty of Health Sciences, Ben-Gurion University of the Negev, Be’er-Sheva, Israel; 2 Research Department, Tikun Olam LTD, Tel Aviv-Yafo, Israel; 3 Institute for Drug Research, School of Pharmacy, The Hebrew University of Jerusalem, Jerusalem, Israel; 4 Negev Autism Centre, Ben-Gurion University of the Negev, Beer Sheva, Israel; 5 Soroka University Medical and Faculty of Health Sciences, Ben-Gurion University of the Negev, Beer Sheva, Israel

## Abstract

There has been a dramatic increase in the number of children diagnosed with autism spectrum disorders (ASD) worldwide. Recently anecdotal evidence of possible therapeutic effects of cannabis products has emerged. The aim of this study is to characterize the epidemiology of ASD patients receiving medical cannabis treatment and to describe its safety and efficacy. We analysed the data prospectively collected as part of the treatment program of 188 ASD patients treated with medical cannabis between 2015 and 2017. The treatment in majority of the patients was based on cannabis oil containing 30% CBD and 1.5% THC. Symptoms inventory, patient global assessment and side effects at 6 months were primary outcomes of interest and were assessed by structured questionnaires. After six months of treatment 82.4% of patients (155) were in active treatment and 60.0% (93) have been assessed; 28 patients (30.1%) reported a significant improvement, 50 (53.7%) moderate, 6 (6.4%) slight and 8 (8.6%) had no change in their condition. Twenty-three patients (25.2%) experienced at least one side effect; the most common was restlessness (6.6%). Cannabis in ASD patients appears to be well tolerated, safe and effective option to relieve symptoms associated with ASD.

## Introduction

There has been a 3-fold increase during the last 3 decades in the number of children diagnosed with autism spectrum disorders worldwide^1–5^. No specific treatments are currently available and interventions are focussing on lessening of the disruptive behaviors, training and teaching self-help skills for a greater independence^6^.

Recently, CBD enriched cannabis has been shown to be beneficial for children with autism^7^. In this retrospective study on 60 children, behavioural outbreaks were improved in 61% of patients, communication problems in 47%, anxiety in 39%, stress in 33% and disruptive behaviour in 33% of the patients. The rationale for this treatment is based on the previous observations and theory that cannabidiol effects might include alleviation of psychosis, anxiety, facilitation of REM sleep and suppressing seizure activity^8^. A prospective single-case-study of Dronabinol (a THC-based drug) showed significant improvements in hyperactivity, lethargy, irritability, stereotypy and inappropriate speech at 6 month follow-up^9^. Furthermore, Dronabinol treatment of 10 adolescent patients with intellectual disability resulted in 8 patients showing improvement in the management of treatment-resistant self-injurious behaviour^10^.

In 2007, The Israel Ministry of Health began providing approvals for medical cannabis, mainly for symptoms palliation. In 2014, The Ministry of Health began providing licenses for the treatment of children with epilepsy. After seeing the results of cannabis treatment on symptoms like anxiety, aggression, panic, tantrums and self-injurious behaviour, in children with epilepsy, parents of severely autistic children turned to medical cannabis for relief.

Although many with autism are being treated today with medical cannabis, there is a significant lack of knowledge regarding the safety profile and the specific symptoms that are most likely to improve under cannabis treatment. Therefore, the aim of this study was to characterize the patient population receiving medical cannabis treatment for autism and to evaluate the safety and efficacy of this therapy.

## Results

### Patient population

During the study period, 188 ASD patients initiated the treatment. Diagnosis of ASD was established in accordance with the accepted practice in Israel; six board certified paediatric psychiatrists and neurologists were responsible for treatment of 125 patients (80.6%), the remaining 30 children were referred by 22 other physicians. Table 1 shows demographic characteristics of the patient population. The mean age was 12.9 ± 7.0 years, with 14 (7.4%) patients being younger than the age of 5, 70 patients (37.2%) between 6 to 10 years and 72 (38.2%) aged 11 to 18. Most of the patients were males (81.9%). Twenty-seven patients (14.4%) suffered from epilepsy and 7 patients (3.7%) from Attention Deficit Hyperactivity Disorder (ADHD).

**Table 1:** Demographic and clinical characteristics of patients at intake.

	Total (188)
Mean age (SD)	12.9 (7.0)
Gender (male), No. (%)	154 (81.9)
Mean body mass index (SD)	29.0 (5.3)
Previous experience with cannabis (Yes), No. (%)	19 (10.1)
**Comorbidities:**	
Epilepsy, No. (%)	27 (14.4)
Attention Deficit Hyperactivity Disorder, No. (%)	7 (3.7)
Tourette syndrome, No. (%)	4 (2.1)
Celiac Disease, No. (%)	3 (1.6)
Anxiety Disorder, No. (%)	3 (1.6)

At baseline parents of 188 patients reported on average of 6.3 ± 3.2 symptoms. Table 2 shows the prevalence of symptoms with most common being restlessness (90.4%), rage attacks (79.8%) and agitation 78.7%.

**Table 2:** Symptom prevalence and change.

	Intake prevalence Total (188)	Change at six months		
		Symptom disappeared	Improvement	No change or deterioration
Restlessness, No. (%)	170 (90.4)	1 (1.2)	71 (89.8)	7 (8.8)
Rage attacks, No. (%)	150 (79.8)	1 (1.3)	65 (89.0)	7 (9.5)
Agitation, No. (%)	148 (78.7)	1 (1.4)	57 (83.8)	10 (14.7)
Sleep problems, No. (%)	113 (60.1)	9 (19.5)	27 (58.6)	10 (21.7)
Speech Impairment, No. (%)	113 (60.1)	—	15 (30)	35 (70)
Cognitive impairment, No. (%)	91 (48.4)	—	15 (27.2)	40 (72.7)
Anxiety, No. (%)	69 (36.7)	—	24 (88.8)	3 (11.1)
Incontinence, No. (%)	51 (27.1)	2 (9.0)	7 (31.8)	13 (59.0)
Seizures, No. (%)	23 (12.2)	2 (15.3)	11 (84.6)	—
Limited Mobility, No. (%)	17 (9.0)	2 (18.1)	—	9 (81.8)
Constipation, No. (%)	15 (8.0)	1 (12.5)	6 (62.5)	2 (25)
Tics, No. (%)	15 (8.0)	1 (20.0)	4 (80.0)	—
Digestion Problems, No. (%)	14 (7.4)	1 (12.5)	5 (62.5)	2 (25.0)
Increased Appetite, No. (%)	14 (7.4)	1 (33.3)	1 (33.3)	1 (33.3)
Lack of Appetite, No. (%)	14 (7.4)	2 (40.0)	1 (20.0)	2 (40.0)
Depression, No. (%)	10 (5.3)	—	5 (100.0)	—

Symptom prevalence at intake in 188 patients assessed at intake and change at six months in patients responding to the six-month questionnaire.

Cannabis products recommended to the patients were mainly oil applied under the tong (94.7%). Seven patients (3.7%) received a license to purchase oil and inflorescence and three patients (1.5%) received a license to purchase only inflorescence. Most patients consumed oil with 30% CBD and 1.5% THC, on average 79.5 ± 61.5 mg CBD and 4.0 ± 3.0 mg THC, three times a day (for a more detailed distribution of CBD/THC consumptions see Supplementary Fig. S1). Insomnia recorded in 46 patients (24.4%) was treated with an evening does of 3% THC oil with on average additional 5.0 ± 4.5 mg THC daily. All the products content was validated by HPLC (High Performance Liquid Chromatography) in each production cycle. The cannabis dose was not significantly associated with weight (r correlation coefficient = −0.13, p = 0.30), age (r correlation coefficient = −0.10, p = 0.38), or gender (p = 0.38).

### Follow-up, one month

**Figure 1:**
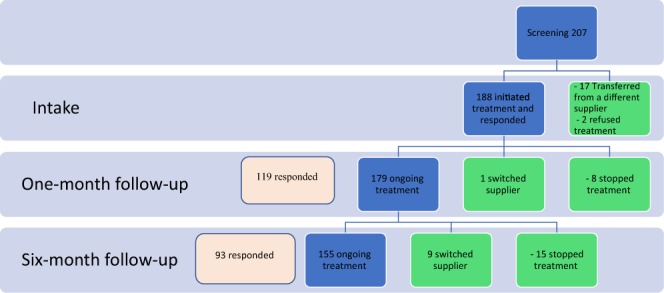
The study population in the three follow-up periods, at intake, after one month and after six months of medical cannabis treatment.

After one month, out of 188 patients, 8 (4.2%) stopped treatment, 1 (0.5%) switched to a different cannabis supplier, and 179 patients (94.6%) continued active treatment (Fig. 1). Of the latter group, 119 (66.4%) responded to the questionnaire with 58 patients (48.7%) reporting significant improvement, 37 (31.1%) moderate improvement; 7 patients (5.9%) experienced side effects and 17 (14.3%) reported that the cannabis did not help them.

The reported side effects at one month were: sleepiness (1.6%), bad taste and smell of the oil (1.6%), restlessness (0.8%), reflux (0.8%) and lack of appetite (0.8%).

### Follow-up, six months

After six months, of the 179 patients assessed in the one-month follow-up, 15 patients (8.3%) stopped treatment, 9 (4.9%) switched to a different cannabis supplier and 155 patients (86.6%) continued treatment (Fig. 1). Of the latter group, 93 (60.0%) responded to the questionnaire with 28 patients (30.1%) reporting a significant improvement, 50 patients (53.7%) moderate improvement, 6 patients (6.4%) slight improvement and 8 (8.6%) having no change in their condition. None of the variables entered to the multivariate analysis to predict treatment success was statistically significant.

To assess the potential response bias, we have compared baseline characteristics between 93 respondents and 62 non-respondents to the 6-month questionnaire. The former group was slightly older (13.7 ± 0.8 vs. 10.8 ± 0.5, p = 0.004).

### Quality of Life

Quality of life, mood and ability to perform activities of daily living were assessed before the treatment and at six months. Good quality of life was reported by 31.3% of patients prior to treatment initiation while at 6 months good quality of life was reported by 66.8% (p < 0.001, Supplementary Fig. S2). Positive mood was reported by the parents on 42% before treatment and 63.5% after 6 months of treatment (p < 0.001). The ability to dress and shower independently was significantly improved from 26.4% reported no difficulty in these activities prior to the treatment to 42.9% at six months (p < 0.001). Similarly, good sleep and good concentration were reported by 3.3% and 0.0% (respectively) before the treatment and on 24.7% (p < 0.001) and 14.0% (p < 0.001) during an active treatment (Table 3).

**Table 3:** Assessment of daily activities.

	Sleep			Eating with Appetite			Concentration on daily tasks			Bowel Activity		
	Before	During	p value	Before	During	p value	Before	During	p value	Before	During	p value
Severe difficulty	44 (47.3)	2 (2.2)	<0.001	2 (2.2)	1 (1.1)	0.751	75 (80.6)	21 (22.6)	<0.001	3 (3.2)	2 (2.2)	0.242
Moderate difficulty	18 (19.4)	27 (29.0)		6 (6.5)	13 (14.0)		11 (11.8)	41 (44.1)		13 (14.0)	17 (18.3)	
No difficulty	28 (30.1)	39 (41.9)		59 (63.4)	47 (50.5)		2 (2.2)	11 (11.8)		71 (76.3)	54 (58.1)	
Good	2 (2.2)	15 (16.1)		10 (10.8)	16 (17.2)		0	10 (10.8)		5 (5.4)	13 (14.0)	
Very Good	1 (1.1)	8 (8.6)		16 (17.2)	14 (15.1)		0	3 (3.2)		1 (1.1)	4 (4.3)	

Ability to perform activities of daily living was assessed prior to and six months after initiation of cannabis treatment. Numbers in parenthesis represent the % of patients.

The improved symptoms at 6 months included seizures, of the 13 patients on an active treatment at six months 11 patients (84.6%) reported disappearances of the symptoms and two patients reported improvement; restlessness and rage attacks were improved in 72 patients (91.0%) and 66 (90.3%) respectively (Table 2).

### Medications Use

The most common concomitant chronic medications on the intake were antipsychotics (56.9%), antiepileptics (26.0%), hypnotics and sedatives (14.9%) and antidepressants (10.6%). Out of 93 patients responding to the follow-up questionnaire, 67 reported use of chronic medications at intake. Overall, six patients (8.9%) reported an increase in their drugs consumption, in 38 patients (56.7%) drugs consumption remained the same and 23 patients (34.3%) reported a decrease, mainly of the following families: antipsychotics, antiepileptics antidepressants and hypnotics and sedatives (Table 4). Antipsychotics, the most prevalent class of medications taken at intake (55 patients, 33.9%); at 6 months it was taken at the same dosage by 41 of them (75%), 3 patients (5.4%) decreased dosage and 11 patients (20%) stopped taking this medication (Table 4).

**Table 4:** Concomitant medications.

Medication family	Intake	Change at six months follow-up				
	Total	Stopped taking this medication	Dosage decreased	Has not changed	Dosage increased	New medication
Antipsychotics, n (%)	55	11 (20)	3 (5)	41 (75)	0	0
Antiepileptics, n (%)	46	6 (13)	0	35 (76)	2 (4.5)	3 (6.5)
Antidepressants, n (%)	10	3 (30)	0	4 (40)	1 (10)	2 (20)
Hypnotics and sedatives, n (%)	10	2 (20)	1 (10)	7 (70)	0	0
Anxiolytics, n (%)	7	2 (28)	0	5 (72)	0	0

Concomitant medications use at the baseline and six months follow up in patients responding to the six-month questionnaire.

### Side Effects

The most common side effects, reported at six months by 23 patients (25.2%, with at least one side effect) were: restlessness (6 patients, 6.6%), sleepiness (3, 3.2%), psychoactive effect (3, 3.2%), increased appetite (3, 3.2%), digestion problems (3, 3.2%), dry mouth (2, 2.2%) and lack of appetite (2, 2.2%).

Out of 23 patients who discontinued the treatment, 17 (73.9%) had responded to the follow-up questionnaire at six months. The reasons for the treatment discontinuation were: no therapeutic effect (70.6%, twelve patients) and side effects (29.4%, five patients). However, 41.2% (seven patients) of the patients who discontinued the treatment had reported on intentions to return to the treatment.

## Discussion

Cannabis as a treatment for autism spectrum disorders patients appears to be well-tolerated, safe and seemingly effective option to relieve symptoms, mainly: seizures, tics, depression, restlessness and rage attacks. The compliance with the treatment regimen appears to be high with less than 15% stopping the treatment at six months follow-up. Overall, more than 80% of the parents reported at significant or moderate improvement in the child global assessment.

The exact mechanism of the cannabis effects in patients with ASD is not fully elucidated. Findings from ASD animal models indicate a possible dysregulation of the endocannabinoid (EC) system^11–16^ signalling behaviours, a dysregulation that was suggested to be also present in ASD patients^17^. Mechanism of action for the effect of cannabis on ASD may possibly involve GABA and glutamate transmission regulation. ASD is characterized by an excitation and inhibition imbalance of GABAergic and glutamatergic signalling in different brain structures^18^. The EC system is involved in modulating imbalanced GABAergic^19^ and glutamatergic transmission^20^.

Other mechanism of action can be through oxytocin and vasopressin, neurotransmitters that act as important modulators of social behaviours^21^. Administration of oxytocin to patients with ASD has been shown to facilitate processing of social information, improve emotional recognition, strengthen social interactions, reduce repetitive behaviours^22^ and increase eye gaze^23^. Cannabidiol was found to enhance oxytocin and vasopressin release during activities involving social interaction^16^.

Two main active ingredients (THC and CBD) can have different psychoactive action mechanisms. THC was previously shown to improve symptoms characteristic to ASD patients in other treated populations. For example, patients reported lower frequency of anxiety, distress and depression^24^, following THC administration, as well as improved mood and better quality of life in general^25^. In patients suffering from anxiety, THC led to improved anxiety levels compared to placebo^26^ and in dementia patients, it led to reduction in nocturnal motor activity,violence^27,28^ behavioural and severity of behavioural disorders^29^. Moreover, cannabis was shown to enhances interpersonal communication^30^ and decrease hostile feelings within small social groups^31^.

In our study we have shown that a CBD enriched treatment of ASD patients can potentially lead to an improvement of behavioural symptoms. These findings are consistent with the findings of two double-blind, placebo-controlled crossover studies demonstrating the anxiolytics properties of CBD in patients with anxiety disorder^32,33^. In one, CBD had a significant effect on increased brain activity in the right posterior cingulate cortex, which is thought to be involved in the processing of emotional information^32^, and in the other, simulated public speaking test was evaluated in 24 patients with social anxiety disorder. The CBD treated group had significantly lower anxiety scores than the placebo group during simulated speech, indicating reduction in anxiety, cognitive impairment, and discomfort factors^33^.

The cannabis treatment appears to be safe and side effects reported by the patients and parents were moderate and relatively easy to cope with. The most prevalent side effects reported at six months was restlessness, appearing in less than 6.6% of patients. Moreover, the compliance with the treatment was high and only less than 5% have stopped the treatment due to the side effects. We believe that the careful titration schedule especially in the ASD paediatric population is important for maintaining a low side effects rate and increase of the success rate. Furthermore, we believe that a professional instruction and detailed parents’ training sessions are highly important for the increasing of effect to adverse events ratio.

The present findings should be interpreted with caution for several reasons. Firstly, this is an observational study with no control group and therefore no causality between cannabis therapy and improvement in patients’ wellbeing can be established. Children of parents seeking cannabis therapy might not constitute a representative sample of the patient with the specific disease (self-selection bias). We have not formally confirmed the ASD diagnosis, however all the children included in the study were previously diagnosed with ASD by certified neurologist or psychiatrist, as required by Ministry of Health prior to the initiation of the cannabis-based treatment.

This study was based on a subjective self-report of the patient’s parent’s observation and not by the patients themselves. These reports, with subjective variables such as quality of life, mood, and general effects, may be biased by the parent’s opinion of the treatment. Moreover, even though the effect was assessed at six months, the possibility of the inflated expectations of the novel treatment “miracle” effect cannot be excluded. The questionnaire response rate at 6 months was 60%, thus the estimates of the efficacy and safety of the treatment can be biased. However, high compliance (above 80%) with the treatment provides a good evidence of the patients and parents satisfaction with the treatment.

While this study suggest that cannabis treatment is safe and can improve ASD symptoms and improve ASD patient’s quality of life, we believe that double blind placebo-controlled trials are crucial for a better understanding of the cannabis effect on ASD patients.

## Methods

### Study Population

There are currently over 35,000 patients approved for medical cannabis use in Israel and 15,000 (~42.8%) of them receive treatment at Tikun-Olam Ltd. (TO), the largest national provider of medical cannabis. This study included all patients receiving cannabis license at TO with the diagnosis of autism in the years 2015–2017.

During the routine treatment process at the cannabis clinic, all willing patients underwent an extensive initial evaluation and their health status was periodically assessed by the treating team. At the intake session, the nurse assessed a complete medical history. The patient’s parents were interviewed by the nurse and filled a medical questionnaire, which included the following domains: demographics, comorbidities, habits, concomitant medications, measurements of quality of life and a detailed symptoms check-list. Following intake, the nurse advised on the treatment plan.

### Treatment Regiment

The treatment in majority of the patients was based on cannabis oil (an extract of a high CBD strain dissolve in olive oil in a ratio THC:CBD of 1:20, 30% CBD and 1.5% THC), and underwent an individualized titration. The starting dose was one sublingual drop three times a day with one oil drop (0.05 ml) containing 15 mg CBD and 0.75 mg Δ9-THC. Oil contained 45% olive oil, 30% CBD, 1.5% THC, <1.5% CBC, 0.5% CBG, <0.5% CBDV and <0.1% CBN. The remaining ingredients were terpenes, flavonoids, waxes and chlorophyll

In patients who reported high sensitivity to previously used medications, the treatment started with oil containing 1:20 15% CBD and 0.75% THC. In patients with severe sleep disturbances, following the initial treatment phase, 3% THC oil was added to the evening dose. In cases with a significant aggressive or violent behaviour, 3% THC oil was added.

The dose was increased gradually for each patient depending on the effect of the cannabis oil on the targeted symptoms according to the treatment plan and the tolerability of each patient. Finding of the optimal dose could take up to two months and dosage range is wide: from one drop three times a day to up to 20 drops three times a day of the same product.

After one month, the treating team contacted the parents to follow-up on the treatment progression. At six months patients underwent an additional assessment of the symptom intensity, side effects and quality of life.

### Study outcomes

For safety analysis we have assessed the frequency of the following side effects at one and at six months: physiological effects – headaches, dizziness, nausea, vomiting, stomach ache, heart palpitation, drop in blood pressure, drop in sugar, sleepiness, weakness, chills, itching, red/irritated eyes, dry mouth, cough, increased appetite, blurred vision, slurred speech; cognitive side effects – restlessness, fear, psycho-active effect, hallucinations, confusion and disorientation, decreased concentration, decreased memory or other. The patient parents were asked to provide details of the incidence, duration and severity of the reported side effect.

For the efficacy analysis we used the global assessment approach where the patient parents were asked: “How would you rate the general effect of cannabis on your child condition?” the options were: significant improvement, moderate improvement, slight improvement, no change, slight deterioration, moderate deterioration and significant deterioration. Autism symptoms severity assessment included the following items: restlessness, rage attacks, agitation, speech impairment, cognitive impairment, anxiety, incontinence, depression and more. Quality of life was assessed on a Likert scale ranging from very poor to poor, neither poor nor good and good to very good^34^.

The study was approved by Soroka University Medical Centre Ethics Committee and due to the nature of the data analysis based on the routinely obtained clinical data, it was determined that no informed consent is required. All methods were performed in accordance with the relevant institutional and international research guidelines and regulations.

### Statistical analysis

Continuous variables with normal distribution were presented as means with standard deviation. Ordinary variables or continuous variables with non-normal distribution were presented as medians with an interquartile range (IQR). Categorical variables were presented as counts and percent of the total.

We used t-test and paired t-test for the analysis of the continuous variables with normal distribution. The non-parametric Mann-Whitney U test and paired Wilcoxon test was used whenever parametric assumptions could not be satisfied.

We utilized logistic regression for the multivariate analysis of factors associated with treatment success. We have included the following variables into the models based on clinical considerations: age, gender, number of chronic medications, number of total symptoms, and the three most prevalent symptoms: restlessness, rage attacks and agitation (as a dichotomous variable- yes/no), as reflected in the intake form.

P value < 0.05 was considered to be statistically significant. All analyses were performed at the Clinical Research Centre, Soroka University Medical Centre, Beer-Sheva, Israel using IBM SPSS version 22 (SPSS, Chicago, IL).

### Declarations

The study was approved by Soroka University Medical Center Ethics Committee (study number: SCRC-0415-15) and the need for informed consent was waived due to the retrospective nature of the data analysis.

## Supplementary information

Supplementary figure S1: Distribution of cannabinoids consumptions. Supplementary figure S2: Quality of life assessment.

## Data Availability

The data set generated and/or analysed during the current study are not publicly available due to medical confidentiality but are available from the first author on reasonable request summarized form pending the approval of the IRB.
