# Supplementary material for: Real life Experience of Medical Cannabis Treatment in Autism: Analysis of Safety and Efficacy
Source: Sci Rep. 2019 Jan 17;9:200. doi: 10.1038/s41598-018-37570-y (PMC6336869; doi:10.1038/s41598-018-37570-y)

REAL LIFE EXPERIENCE OF MEDICAL CANNABIS TREATMENT IN AUTISM: ANALYSIS OF SAFETY AND EFFICACY.

Lihi Bar-Lev Schleider MA[1,2], Raphael Mechoulam PhD[3], Naama Saban[2], Gal Meiri MD MHA[4,5], *Victor Novack MD PhD[1].

[1] Clinical Cannabis Research Institute, Soroka University Medical Center and Faculty of Health Sciences, Ben-Gurion University of the Negev, Be'er-Sheva, Israel.

[2] Research Department, Tikun Olam LTD.

[3] Institute for Drug Research, School of Pharmacy, the Hebrew University of Jerusalem, Israel.

[4] Negev Autism Centre, Ben-Gurion University of the Negev, Beer Sheva, Israel

[5] Soroka University Medical and Faculty of Health Sciences, Ben-Gurion University of the Negev, Beer Sheva, Israel

Corresponding author – Victor Novack - VictorNo@clalit.org.il

Supplementary figure S1: Distribution of cannabinoids consumptions. Total CBD (in mg) consumed at every intake by 66 patients receiving oil with 30% CBD and 1.5% THC. THC is 1/20 of the amount of CBD.

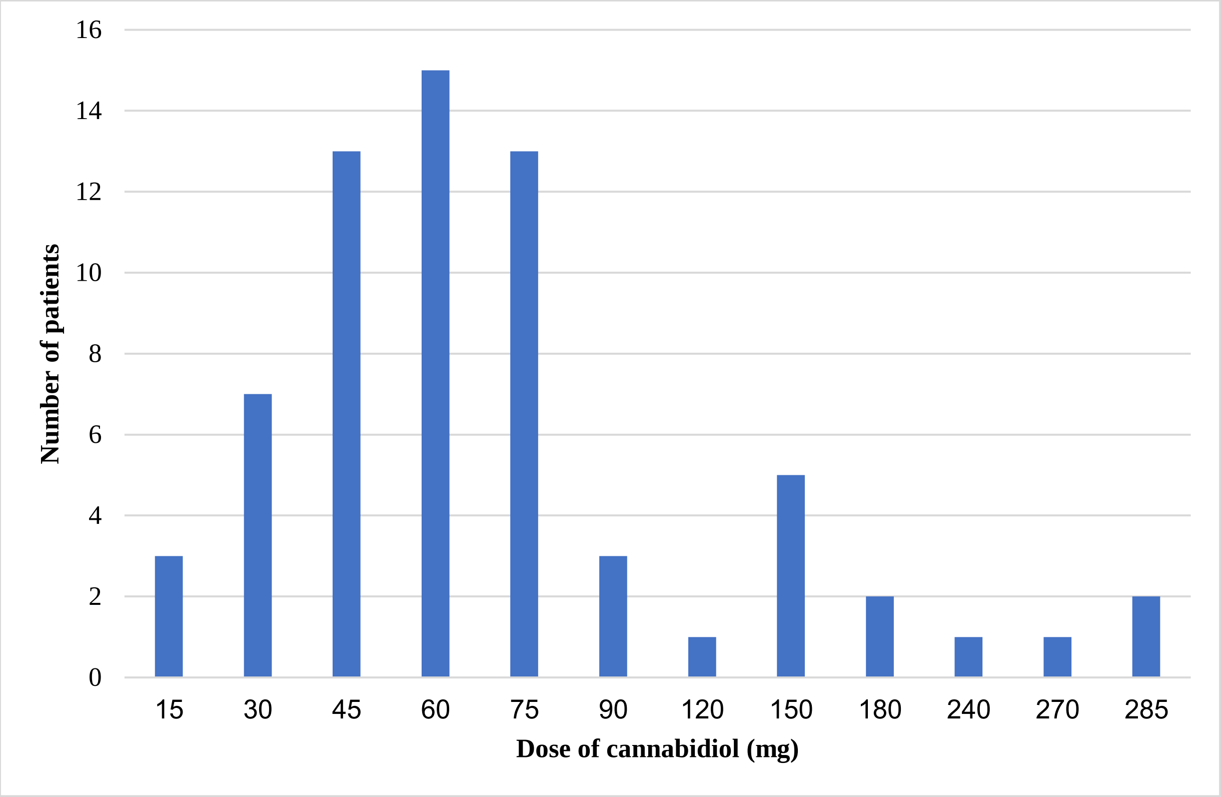

Supplementary figure S2: Quality of life assessment. Quality of life was assessed prior to and six months after initiation of cannabis treatment. $p<0.001$

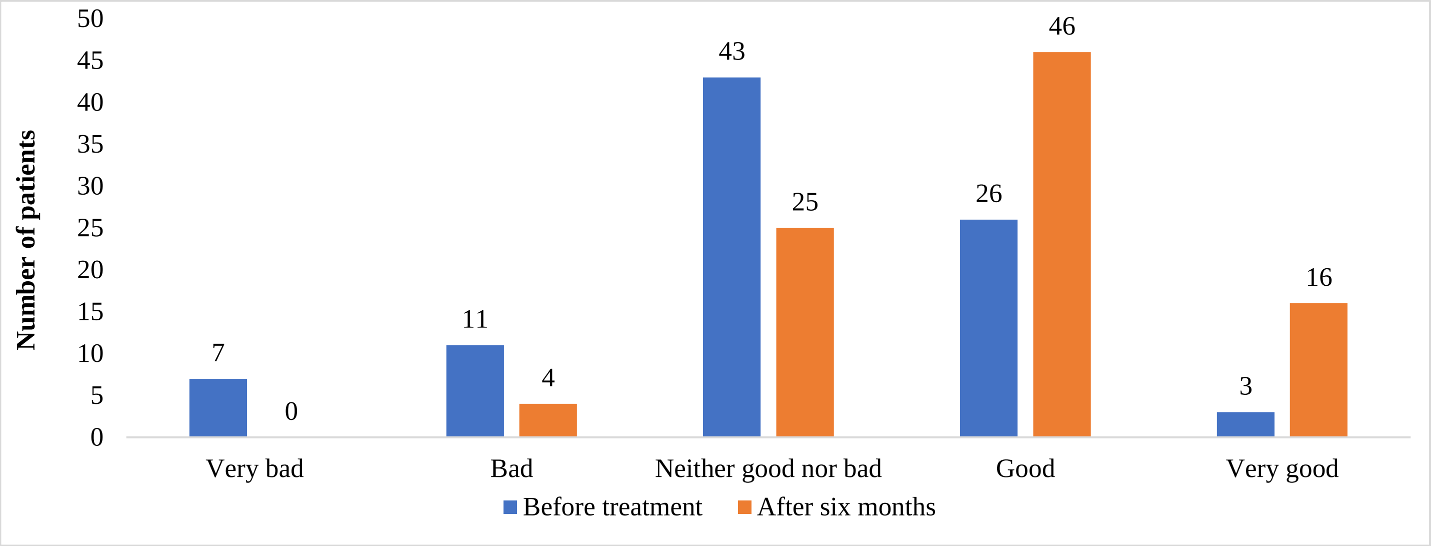

Supplement: Supplementary file 1 — Supplementary figure S1: Distribution of cannabinoids consumptions. Supplementary figure S2: Quality of life assessment. [file 41598_2018_37570_MOESM1_ESM.pdf]
